# Sealing efficacy of system B versus Thermafil and Guttacore obturation techniques evidenced by scintigraphic analysis

**DOI:** 10.4317/jced.52889

**Published:** 2017-01-01

**Authors:** Manuel Marques-Ferreira, Margarida Abrantes, Hugo-Diogo Ferreira, Francisco Caramelo, Maria-Filomena Botelho, Eunice-Virgínia Carrilho

**Affiliations:** 1 PhD, Department of Dentistry, Faculty of Medicine, University of Coimbra, Portugal; 2 PhD, Department of Biophysics and Biomathematics, IBILI-Faculty of Medicine, University of Coimbra, Portugal; 3 DDS, Department of Dentistry, Faculty of Medicine, University of Coimbra, Portugal

## Abstract

**Background:**

This study compared root canal sealing ability, filled by Continuous Wave compaction and two carrier-based obturation systems, using the nuclear medicine approach.

**Material and Methods:**

Fifty-five single-rooted extracted teeth were selected. The crowns were sectioned and each tooth was instrumented using rotary Protaper® Universal system. The roots were divided into 3 experimental groups and two control groups. Forty-five root canals were filled, using Continuous Wave, GuttaCore or Thermafil system and TopSeal sealer. Ten teeth were used as control. On the 7th days the apices were submersed in a solution of sodium pertechnetate 99mTc for 3 hours and the radioactivity was counted.

**Results:**

Although apical leakage in the Continuous Wave group was lower compared with GuttaCore and Thermafil groups, there was no statistical difference (*p*>0.05).

**Conclusions:**

System B, GuttaCore and Thermafil techniques showed a similar sealing effect.

** Key words:**Continuous wave compaction, Gutta percha core-carrier, leakage, nuclear medicine.

## Introduction

The first stage of root canal treatment is shaping, cleaning and disinfection of the root canal system for microbial control. The final step is root canal filling that must fill the space completely with isthmus, deltas and lateral canals and seal the canal to prevent coronal, lateral and apical leakage and recolonization of bacteria which affects the long-term results of endodontic treatment (1,2).

The most commonly used material for root canal obturation is gutta-percha combined with a sealer, because gutta-percha is considered an impermeable material even though it does not bond to the root dentin walls.

Lateral condensation of gutta-percha remains the technique most used worldwide, and is accepted to fill the root canal system. However, studies have shown that this technique fails to give a good seal of the root canal (3).

To solve this problem, many efforts have been made to develop new obturation techniques.

Warm vertical compaction is a technique in which plasticized gutta-percha can be condensed into roots, and is able to create a homogeneous obturation with good tridimensional sealing (4,5).

In attempt to simplify the warm vertical compaction technique, new devices such as BeeFill 2 in 1 (VDW, Munich, Germany), which includes down pack and backfilling equipment in one unit, have been developed.

Despite the development of these new devices, this technique is time-consuming and could create extrusion of the material into periapical tissues (5).

To attempt easier and more reliable filling techniques, several variants of warm gutta-percha condensation have been developed.

The Thermafil system is a carrier-based technique with warm gutta-percha, which is adaptable to the dentinal walls, giving a good seal. This method is a simple and effective procedure that significantly reduces working time while promising high-quality obturation, mainly in narrow and anatomically complex root canals (6,7).

The Thermafil system is criticized for the difficulty in post space preparation and the risks of making ledges or lateral root perforations during this procedure and if nonsurgical root canal retreatment is necessary.

A recent carrier of gutta-percha material, the GuttaCore system, claims that it is able to create a three-dimensional root canal obturation of root canal systems. The carriers of GuttaCore are made from cross-linked gutta-percha that makes it easier to remove for post space preparation and in cases where retreatment is required.

To date, several studies to evaluate the outcomes of different root canal sealers with various leakage models have been used. The major problem of most laboratory-based leakage testing models is that the obtained data are qualitative rather than quantitative, raising doubts about their reliability (8-11).

The use of sodium pertechnetate (99mTcNaO4) in nuclear medicine is well established and the evolution of diagnostic conventional nuclear medicine can be mainly attributed to the existence and the chemical versatility of this radionuclide (Pogrel *et al.* 1995, Sharmila *et al.* 2001), (12,13).

Considering all the characteristics of 99mTc and considering that Nuclear Medicine is an approach with high sensitivity and specificity, radionuclides may provide quantitative and objective results concerning infiltration.

Thus, the aim of this study was to use nuclear medicine methodologies to assess microleakage of root canals. For that purpose we used 99mTcNaO4 to compare the sealing ability of roots filled with Continuous Wave, GuttaCore or Thermafil, with those that were filled with gutta-percha and TopSeal, an epoxy-based root canal sealer.

## Material and Methods

Fifty-five extracted human teeth, with a single root and the apex completely formed, were used in this study. The study was approved by the ethics committee of the Faculty of Medicine University of Coimbra-PT. To prevent bacterial growth, these teeth were stored at 4ºC until use in a 0.9% sodium chloride solution containing 0.02% sodium azide.

The crowns were removed with a high-speed bur and water spray, in order to obtain roots approximately 15 mm long. Canal length was determined by inserting a K file, ISO size #15 (Dentsply Maillefer, CH-1338 Ballaigues, Switzerland) through the apical foramen and the working length was established 1 mm short of that length.

Root canal preparation was performed using ProTaper Universal® nickel-titanium rotary instruments (Dentsply Maillefer, CH-1338 Ballaigues, Switzerland). The handpiece was used with an electric engine (X-Smart; Dentsply Maillefer, CH-1338 Ballaigues, Switzerland) at 300 rpm and 3 Ncm. Instrumentation was completed with F3 instruments up to the working length. After the use of each instrument, the canals were irrigated with 3 mL of 2.5% NaOCl using a 27-gauge Monoject irrigation needle (Sherwood Medical, St. Louis, MO). The final rinse was performed using 3 mL of 2.5% NaOCl for 3 min and ml of 17% EDTA for 3 min (Pulpdent Corporation, Watertown, MA), followed by 3 mL of saline solution for 1 min to neutralize the EDTA. At the end, the samples were dried with F3 paper points (Dentsply Maillefer, CH-1338 Ballaigues, Switzerland) and randomly divided into 3 experimental groups of 15 teeth each (Groups 1, 2 and 3) and two control groups of 10 teeth.

In Group 1 (Topseal/GuttaCore), the obturations were performed with TopSeal® sealer placed in the canal using 30.04 master points. Then, a GuttaCore™ Obturator #30 was inserted into the canal after being thermo-plasticized in the ThermaPrep oven (Dentsply Maillefer, CH-1338 Ballaigues, Switzerland), according to the manufacturer’s instructions. The carrier was twisted off, and the excess of gutta-percha was removed with a hot instrument.

In Group 2 (Topseal/ Thermafil® ), the obturations were performed using Thermafil® Obturators F3 and placing TopSeal® sealer into the canal which had been thermo-plasticized in the ThermaPrep oven (Dentsply Maillefer, CH-1338 Ballaigues, Switzerland), according to the manufacturer’s instructions. After the filling procedures, the carrier was cut with a round bur at the orifice entrance, and then the excess of gutta-percha was removed.

In Group 3 (TopSeal/Continuous Wave), the root canal obturation was carried out using a Beefill 2in1® device (VDW, München, Germany) with the continuous wave technique. An F3 gutta-percha point (Dentsply Maillefer, CH-1338 Ballaigues, Switzerland) and TopSeal® as a sealer was introduced into the canal. A plugger in a Beefill 2in1® device heat source was heated to 180ºC and introduced into the canal 5 mm short of the working length. After that a plugger 1-2 (Maillefer, Ballaigues, Switzerland) was placed in the canal and vertically pressed to compact the apical master cone gutta-percha. The middle and coronal thirds of the canal were completely filled with Beefill Backfill of hot gutta-percha and down-packed with a plugger.

After the filling procedures, the root canal orifices were sealed using a flowable resin composite (Versite Flow, Kerr SA, 6934 Bioggio, Switzerland) and 2 radiographs were taken in orthoradial and proximal views to analyse the quality of the canal filling.

All experimental procedures were carried out by the same endodontist.

The filled root segments were stored for 1 week at 37°C and 100% relative humidity to allow the sealers to set completely, before leakage evaluation with a nuclear medicine approach.

In Group 4 (positive control), the procedures were the same as those described for the experimental group except that the prepared root canal space was not filled.

The samples of the experimental and positive control groups were covered by two layers of nail varnish, except for 2 apical mm. In the negative control group (Group 5), the root surface was completely sealed with nail varnish, including the apical foramen. The teeth were immersed floating for three hours in an Eppendorf containing a solution of 99mTcNaO4. After 3 hours the varnish was removed and the scintigraphic images were acquired for each tooth using a gamma camera (GE 400 AC, Milwaukee, USA). For each tooth a static image was acquired for three minutes for a 512x512 matrix size regions of interest (ROIs) in each image were drawn over each tooth, to obtain the total counts and the counts per minute (cpm). All the nuclear medicine procedures were carried out by one nuclear medicine specialist, in a blind method.

The statistical analyses were performed using statistics software ‘R - Statistical Data Analysis v.2.15.0’ and statistical significance was assessed using α = 0.05. Kruskal-Wallis analysis was carried out to compare differences between the groups.

## Results

The leakage in the experimental and control groups are presented in  Table 1 as mean and standard deviation.

**Table 1:**
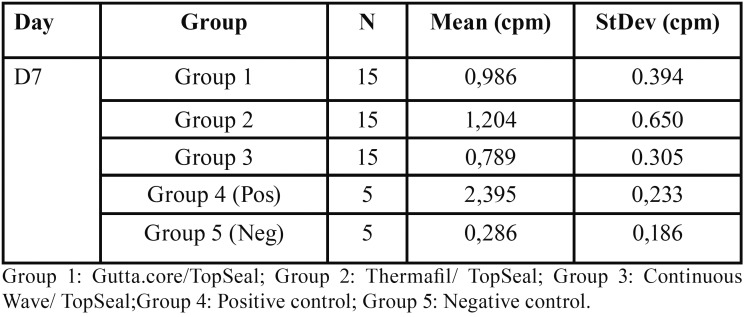
Evaluation of leakage in counts per minute (cpm) according the obturation techniques.

The highest leakage in all experimental groups was observed in the Thermafil system (G2=1.204 cpm) followed by the GuttaCore (G1=0.986 cpm) and Continuous Wave Technique (G3=0.789 cpm) ( Table 1).

Although in Group 3, (Continuous Wave/TopSeal), the number of counts was less than in the group 1 and group 2, these differences are not statistically significant (*p*=0.254).

Comparing the experimental groups with the negative control (0.286 cpm), there was statistically significant differences (*p*= 00).

Comparing the experimental groups with the positive control group (2.395 cpm), there was statistically significant differences (*p*= 00).

## Discussion

The aim of root canal filling is to obtain an effective sealing of the root canal system both coronal and apical and it is a very challenging step in endodontic treatment.

In recent years, new materials and sealers for obturation have been developed. In the carrier system, gutta-percha material is deposited periferically on a plastic carrier and has been widely used, but there are a claim that is difficult to remove when retreatment is needed or post-placement is required. In a recent advance in obturation materials, Dentsply introduced a new carrier system with GuttaCore, a cross-linked gutta-percha core obturator without a plastic core, making post-placement and retreatment easier.

The analysis of the sealing ability of new root canal obturation systems is important in both coronal and apical leakage, because they have been cited as a significant cause of post-treatment disease (14,15).

To evaluate the sealing ability of root canal sealers several methods are used. Although dye penetration along the root canal is the most prevalent method used to evaluate the sealing ability of root canal sealers, it is not sufficiently reliable or sensitive (16,17).

Even though bacterial leakage and the fluid transport model have been proven to be more sensitive and clinically more relevant compared with the conventional dye penetration, radionuclide methods are not usually used (14,18,19).

The aim of this study was to use nuclear medicine to compare root canal sealing ability when filled by gutta-percha/TopSeal, using Continuous Wave compaction with two carrier-based obturation/TopSeal systems.

The aim of this study was to compare root canal sealing ability when filled by gutta-percha/TopSeal, via Continuous Wave compaction or with two carrier-based obturation/TopSeal systems, using nuclear medicine.

The most relevant properties of 99mTc consist of its 140 keV gamma photon emission with 89% abundance, which is optimum for imaging with the gamma cameras used in nuclear medicine. Moreover, its half-life of 6 hours is sufficient to prepare radiopharmaceuticals, perform their quality control, and administer them to the patient for imaging studies, while also having a favourable dosimetry. The rapid growth in this field in the last few decades is attributable, in addition to its ideal radionuclide characteristics, to the design and development of 99Mo/99mTc generators and lyophilized kits to facilitate the formulation of 99mTc compounds in hospital radiopharmacies (13). 99mTcNaO4 is obtained in the form of sodium pertechnetate directly from the generator after elution with saline. The reason for our taking this option and the principal advantages of using a radioactive analysis with 99mTcNaO4 is that this radionuclide method is non-destructive, and is a quantitative method that enables measurement of microleakage from the same specimens at intervals over extended periods.

The thermal effect produced by warm gutta-percha obturation on the host tissues is still not clear. Furthermore, the physical and chemical properties of AH Plus were negatively affected by the changes in temperature and also lack uniform heating along the entire length of the spreader (20).

Thermafil is an easy obturation method with short execution time, and according to the manufacturers, its usage gives a good canal seal. According to some authors, obturation with carriers systems has a smaller deviation in leakage values compared with vertical and lateral condensation, which may be a good indication to provide a correct method for clinical use (21).

GuttaCore is an obturation carrier-system method made entirely of gutta-percha with a core obturator prepared with cross-linked gutta-percha. This method of obturation is simple to use and claims to have high-quality and three dimensional root canal filling, with significantly less voids and gaps, than lateral compaction (22).

The carrier, since it is also gutta-percha, can be easily removed from the root canal for post-space preparation and for root filling removal, in those cases where retreatment is required (23).

Under the experimental conditions of the current *ex vivo* experiment, the results demonstrated that the newly developed GuttaCore carrier system in combination with root canal sealer does not improve the microleakage resistance compared with Thermafil/Topseal filling and Continuous Wave compaction technique. Nevertheless, further investigation of other features of root canal sealers is required.

Within the limitations of the present study, the apical sealing efficiency of obturation techniques measured with nuclear medicine testing *in vitro*, was similar.
